# Deep learning systems detect dysplasia with human-like accuracy using histopathology and probe-based confocal laser endomicroscopy

**DOI:** 10.1038/s41598-021-84510-4

**Published:** 2021-03-03

**Authors:** Shan Guleria, Tilak U. Shah, J. Vincent Pulido, Matthew Fasullo, Lubaina Ehsan, Robert Lippman, Rasoul Sali, Pritesh Mutha, Lin Cheng, Donald E. Brown, Sana Syed

**Affiliations:** 1 Rush University Medical Center, Chicago, IL USA; 2 Hunter Holmes McGuire Veterans Affairs Medical Center, Richmond, VA USA; 3 Division of Gastroenterology, Hepatology and Nutrition, Virginia Commonwealth University, Richmond, VA USA; 4 Johns Hopkins University Applied Physics Laboratory, Laurel, MD USA; 5 Division of Gastroenterology, Hepatology, and Nutrition, Department of Pediatrics, University of Virginia School of Medicine, Charlottesville, VA USA; 6 Department of Systems & Information Engineering, University of Virginia, Charlottesville, VA USA

**Keywords:** Image processing, Cancer screening

## Abstract

Probe-based confocal laser endomicroscopy (pCLE) allows for real-time diagnosis of dysplasia and cancer in Barrett’s esophagus (BE) but is limited by low sensitivity. Even the gold standard of histopathology is hindered by poor agreement between pathologists. We deployed deep-learning-based image and video analysis in order to improve diagnostic accuracy of pCLE videos and biopsy images. Blinded experts categorized biopsies and pCLE videos as squamous, non-dysplastic BE, or dysplasia/cancer, and deep learning models were trained to classify the data into these three categories. Biopsy classification was conducted using two distinct approaches—a patch-level model and a whole-slide-image-level model. Gradient-weighted class activation maps (Grad-CAMs) were extracted from pCLE and biopsy models in order to determine tissue structures deemed relevant by the models. 1970 pCLE videos, 897,931 biopsy patches, and 387 whole-slide images were used to train, test, and validate the models. In pCLE analysis, models achieved a high sensitivity for dysplasia (71%) and an overall accuracy of 90% for all classes. For biopsies at the patch level, the model achieved a sensitivity of 72% for dysplasia and an overall accuracy of 90%. The whole-slide-image-level model achieved a sensitivity of 90% for dysplasia and 94% overall accuracy. Grad-CAMs for all models showed activation in medically relevant tissue regions. Our deep learning models achieved high diagnostic accuracy for both pCLE-based and histopathologic diagnosis of esophageal dysplasia and its precursors, similar to human accuracy in prior studies. These machine learning approaches may improve accuracy and efficiency of current screening protocols.

## Introduction

In patients with Barrett’s esophagus (BE) undergoing surveillance endoscopy, high-definition white light endoscopy alone has poor overall sensitivity for dysplasia^1^. In order to increase the likelihood of identifying dysplasia, guidelines recommend the Seattle protocol, which involves taking four-quadrant random biopsies at 1–2 cm intervals^2^. However, this protocol does not permit real-time diagnosis or therapy and is labor-intensive, leading to low adherence^3,4^. Additionally, numerous studies have documented poor inter-observer agreement among pathologists when diagnosing both low-grade^5–7^ and high-grade dysplasia^8^, suggesting significant room for improvement in even the gold standard of histopathologic diagnosis. In fact, rates of progression from low-grade dysplasia to esophageal adenocarcinoma have been estimated as high as 11.4% in one study^9^, and missed cancer diagnosis (diagnosed within one year of endoscopy) rates are as high as 25%^5^.

Probe-based confocal laser endomicroscopy (pCLE) is a novel endoscopic imaging technique that permits real-time in-vivo histologic analysis of esophageal mucosa. It is based on the principle of illuminating a tissue with a low-power laser and detecting the reflected light to provide in-depth images with a resolution of 1 micron, allowing for tissue architecture visualization^10,11^. In BE patients, previously published studies have documented high specificity for pCLE in identifying high-grade dysplasia and esophageal adenocarcinoma^6^. However, even among experts, the sensitivity of this technology for neoplasia is low, reported at 28% when interpreted blinded post-procedure and 12% when performed intra-procedurally^12^. Therefore, pCLE in its current iteration is not a suitable alternative to the Seattle protocol^6^. Additionally, pCLE is plagued by substantial motion artifact and requires specialized training to interpret, limiting its clinical utility^13^.

We suggest deploying deep-learning-based image recognition models, which offer the potential to improve accuracy of pCLE and histopathology and to recognize patterns that may have eluded human visual analysis. Within the field of gastroenterology, investigators (including our group) have applied deep learning imaging models to colonoscopy^14–17^, and capsule endoscopy data^18,19^. Such models have been also applied to whole-slide images of BE biopsies^20^ and to pCLE images to detect motion artifacts^13^.

In this study, we aim to determine the accuracy with which our deep learning models can classify BE and related diseases and indirectly compare these results to prior studies of human diagnostic accuracy. Second, we aim to determine the specific tissue structures on both pCLE and H&E-stained biopsy that the models are using for their decision-making process. Prior work done by this group has demonstrated some success in pCLE interpretation with the novel video deep learning models, but this question of which aspects of the pCLE videos were most relevant to the model remains unanswered^21^.

With regards to classification of biopsy images, there arises a problem specific to deep learning and other computer-based image recognition techniques. Deep-learning-based image recognition requires training a model on a labeled dataset consisting of images with their corresponding class labels in order for the model to learn the characteristics of abnormal tissue^22^. In other words, building these datasets usually involves tedious annotations by humans, which create a high barrier to entry for researchers developing such models. Therefore, we additionally aimed to develop biopsy recognition models that limited the required amount of human annotation.

## Methods

### Participants and data collection

#### pCLE videos

pCLE videos were obtained from two prior prospectively conducted studies in order to maximize data from each tissue class ^6,23^. In one study, patients undergoing surveillance of BE between 2014 and 2016 (Table 1) underwent high-definition white light imaging (HD-WLE) followed by narrow band imaging (NBI). All patients then underwent pCLE using a 2.5 mm gastroflex ultra-high-definition probe (Cellvizio GI system, Mauna Kea, Paris, France) on any areas considered suspicious on HD-WLE or NBI, as well as in 4-quadrants at 1-cm intervals. Details regarding procedure techniques are published elsewhere^6^. The pCLE videos were reviewed in real-time as well as by two experts [TS, PM – practicing gastroenterologists trained in endomicroscopy] blinded to all other patient data including histopathological diagnosis and patient characteristics. Disagreements were resolved by consensus between expert reviewers. Video sequences from a second clinical trial (2016–2018)^23^ were included to enrich the sample for normal squamous epithelium, which was lacking from the BE dataset. In this study, a 3-min pCLE video recording was obtained from each patient 6 cm above the top of the gastric folds in patients with refractory symptoms of gastroesophageal reflux disease (GERD) and asymptomatic controls. The aim of this particular study was to assess whether time to visualize squamous cells on pCLE after visualization of fluorescein correlated with in vitro permeability, and predicted GERD vs. non-GERD. These videos were separated into five-second sequences for our pCLE models as outlined below.

**Table 1:** Basic patient characteristics.

Biopsy Patients							
Variable, mean (SD)	Total (n = 130)*	Train Set (n = 15)	Test Set (n = 11)	Unlabeled Train Set (n = 104)	p-value		
Age, years	65 (8.4)	67.3 (8.7)	67.2 (11.0)	63.9 (8.0)	0.141		
BMI	30.0 (7.0)	28.7 (5.8)	28.7 (3.2)	30.3 (7.5)	0.954		
BE Length, Circumferential, cm	2.1 (3.2)	1.9 (1.9)	3.5 (3.9)	1.9 (3.3)	0.353		
BE Length, Maximal, cm	3.7 (3.2)	4.5 (2.6)	4.6 (3.6)	3.4 (3.3)	0.216		
Duration of BE, years	5 (5.2)	5 (5.0)	8 (6.5)	5 (5.0)	0.340		
Hiatal Hernia Size, cm	2.4 (2.0)	2.7 (2.1)	3.5 (2.5)	2.2 (1.9)	0.323		

Variable, n (%)	Total (n = 130)*	Train Set (n = 15)	Test Set (n = 11)	Unlabeled Train Set (n = 104)	Train vs. Test p-value	Train vs. Unlabeled p-value	Unlabeled vs. Test p-value
Male Gender	125 (96.1%)	15 (100%)	11 (100%)	99 (95.2%)	1.000	0.022	0.022
Caucasian Race	121 (93.1%)	14 (93.3%)	11 (100%)	96 (92.3%)	0.301	0.883	0.003
PPI Use	109 (83.8%)	14 (93.3%)	10 (90.9%)	85 (81.7%)	0.822	0.121	0.332
Current Smoker	30 (23.3%)	4 (26.7%)	1 (9.1%)	25 (24.3%)	0.220	0.844	0.115
Prior Smoker	91 (70.5%)	11 (73.3%)	6 (54.5%)	74 (71.8%)	0.319	0.903	0.269

pCLE Patients							
Variable, mean (SD)	Total (n = 79)	Train Set (n = 68)	Validation Set (n = 65)	Test Set (n = 63)	p-value		
Age, years	63 (8.1)	63 (8.3)	63 (8.3)	63 (8.4)	0.999		
BMI	30.1 (6.8)	29.8 (6.8)	29.7 (6.7)	29.4 (6.5)	0.912		
BE Length, Circumferential, cm	1.7 (3.0)	1.4 (2.4)	1.4 (2.5)	1.4 (2.5)	0.984		
BE Length, Maximal, cm	3.2 (3.1)	2.9 (2.5)	3.0 (2.6)	3.0 (2.5)	0.995		
Duration of BE, years	5 (5.7)	5 (5.6)	5 (5.7)	5 (5.2)	0.934		
Hiatal Hernia Size, cm	2.3 (2.4)	2.1 (2.2)	2.2 (2.2)	2.2 (2.2)	0.994		

Variable, n (%)	Total (n = 79)	Train Set (n = 68)	Validation Set (n = 65)	Test Set (n = 63)	Train vs. Validation p-value	Train vs. Test p-value	Validation vs. Test p-value
Male Gender	70 (90.9%)	62 (93.9%)	60 (93.8%)	59 (96.7%)	0.946	0.454	0.433
Caucasian Race	65 (84.4%)	56 (84.8%)	54 (84.4%)	50 (82.0%)	0.940	0.663	0.719
PPI Use	63 (80.8%)	53 (80.3%)	51 (79.7%)	51 (83.6%)	0.930	0.628	0.571
Current Smoker	24 (37.5%)	23 (42.6%)	22 (41.5%)	22 (43.1%)	0.910	0.955	0.867
Prior Smoker	48 (75.0%)	42 (75.0%)	39 (73.6%)	39 (73.6%)	0.866	0.866	1.000

* Labeled train and test sets comprise a relatively small number of labeled patient data compared to the total (25 of 130 patients). The remaining data (n = 104) were used in the unlabeled training set for the patch-level semi-supervised learning model.

Basic patient characteristics, subdivided by pCLE versus biopsy and by training versus validation versus test set for the models. Note that a given individual patients’ biopsy or pCLE data may have been placed into multiple datasets, but the specific sequences and biopsies in each dataset were unique and without crossover.

#### Histopathology

All patients in the prospective pCLE-BE study^6^ (2014–2016) underwent targeted biopsy or mucosal resection, as well as Seattle protocol biopsies. In an effort to increase the quantity of data, we also included patients who underwent standard of care biopsies during upper endoscopy for BE surveillance from 2016–2019. These patients all underwent HD-WLE, NBI, and acetic acid chromoendoscopy followed by targeted biopsies/mucosal resection, and Seattle protocol biopsies. All biopsy specimens were fixed in formalin. Samples were embedded to exhibit full mucosal thickness. The paraffin blocks were sectioned into vertical sections of 3 microns each to create biopsy slides that were stained with hematoxylin and eosin. All suspected diagnoses of dysplasia or malignancy required consensus of two or more pathologists. Pathology results were prospectively recorded.

### pCLE deep learning video model design

#### pCLE datasets

In order to standardize the length of each pCLE video for analysis, the raw pCLE videos from each patient were divided into five-second sequences (at 24 frames per second, so each sequence contained 120 frames), and those sequences that fell short of the 5 s were padded with blank frames. Each of these sequences was reviewed in a blinded fashion by two trained pCLE interpreters and annotated as one of 3 classes: squamous, Barrett’s (non-dysplastic intestinal metaplasia), and dysplasia/cancer. Disagreements were resolved by a third expert or by consensus. The frames with a high degree of noise (i.e. signals indiscernible by the annotator) were excluded from the dataset. In instances where there are two classes present in one five-second sequence, a protocol was created in which the sequence was labeled with the class of higher severity (i.e. dysplasia > Barrett’s > squamous). For example, if both dysplasia and Barrett’s characteristics are present, then the annotator labeled the clip as “dysplasia.” These reviewed and annotated sequences represent the ground truth in order to calculate metrics of performance. The train/validation/test sets were split at the patient level and were randomly distributed in a 60/20/20 ratio, respectively (Table S1). Further details on the dataset transformation are supplied in the supplement.

#### pCLE video classification models

The primary issue in conducting the analysis of pCLE video sequences with these datasets is that, given the relative rarity of dysplasia compared to non-dysplastic Barrett’s or squamous tissue^24^, the dysplasia class represents less than 4% of the sequences in the complete pCLE dataset (Table S1). In order to circumvent this issue, we developed one model that uses traditional attention layers (Attn) and another that uses class-specific multi-module attention layers (MultiAttn) that would perform better with an imbalanced dataset^21^. The general architecture of the model consists of three sequential modules. First, the frame-level network converts video frames into a 256-dimensional frame representation consisting of 1D embeddings. Second, the pooling network aggregates these frame-level representations into a video-level representation (this is where the Attn and MultiAttn models were employed). Lastly, the video-level representation was used to obtain probabilities of the video sequence belonging to one of the three tissue classes. Further details for these methods are provided in the supplement (Fig. S1).

#### pCLE video model visualization

In order to determine the tissue structures that the model used for its decision-making; we required a tool capable of visualizing the areas of most relevance to the models. Gradient-weighted Class Activation Maps (Grad-CAMs) highlight regions of images that are relevant to the classification of that image^25^. They represent a means of providing model “explainability,” which enables the users to verify the features used by the model for classification, ideally the same features (or new ones) that pathologists use to make the same classification. In order to achieve a visual heat map of the areas of the pCLE frames that were of the highest relevance to the model, we first split the video sequences into individual frames as our Grad-CAMs require still images. We then extracted Grad-CAMs from these single frames of sample sequences from each class, with the sample frames being manually chosen to avoid frames that contained motion artifact and other distractors.

### Biopsy deep learning model design

#### Esophageal biopsy datasets

**Figure 1:**
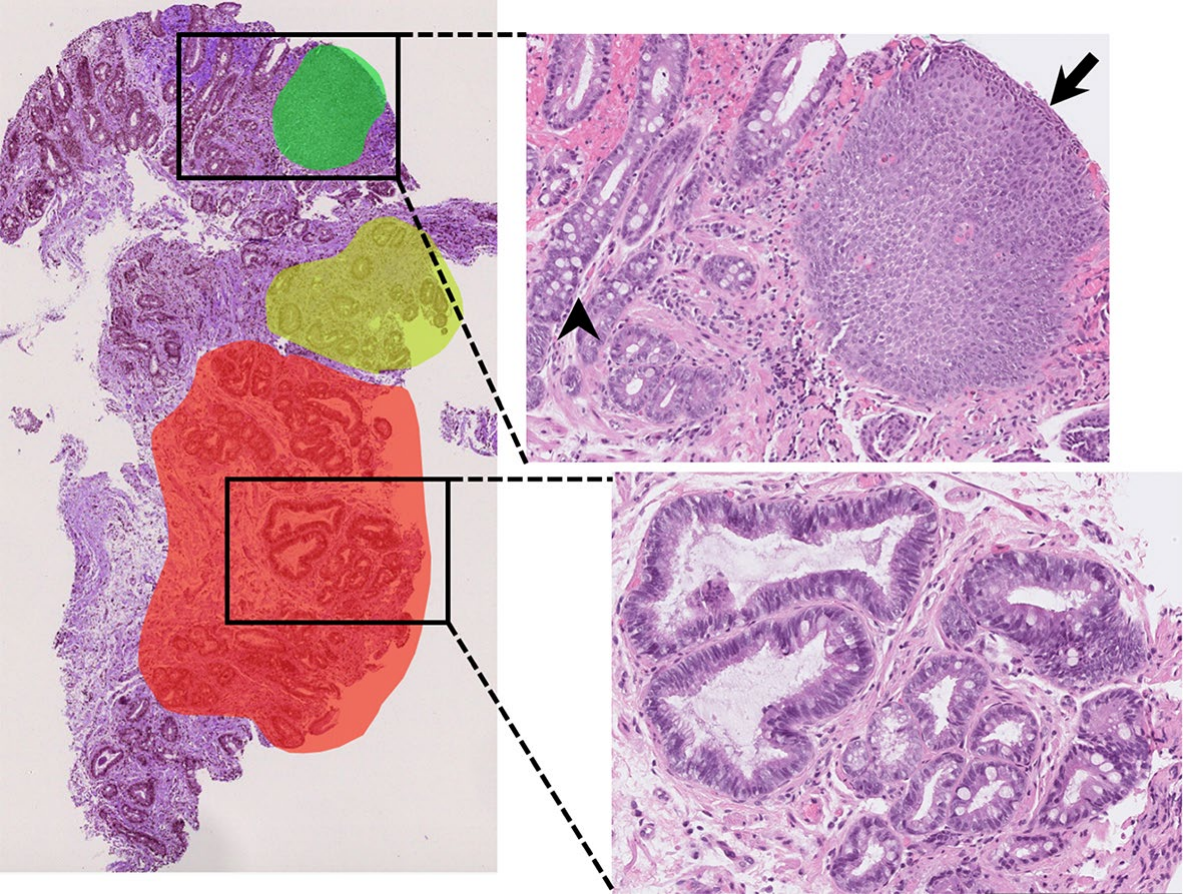
Example of the annotation process on a typical whole-slide image. Red, green, and yellow highlights indicate areas that were annotated and from which labeled patches were taken. Squamous tissue (black arrow), non-dysplastic Barrett’s with Goblet cells (black arrowhead), and dysplastic tissue with crowding and hyperchromasia (lower zoomed section) were all present within the same whole-slide image, demonstrating the value of annotation to avoid model confusion. Created using openslide 1.1.2 (https://openslide.org/api/python/) and PIL 2.2.2 (https://pypi.org/project/Pillow/2.2.2/).

A total of 387 whole-slide images from 130 unique patients were collected. As mentioned in the introduction, a significant issue for deep-learning-based image analysis of whole-slide images is that computers are generally not as adept as humans at quickly scanning a large slide and discovering areas of relevance. In practice, this requires substantial manual annotation of the whole-slide image in order to provide the models with patch-level labels of each class rather than simply labeling the entire whole-slide image with the diagnosis given by the pathologist. As such, a selection of the whole-slide images was manually annotated to highlight examples of each class (squamous, Barrett’s, and dysplasia) within each whole-slide image (Fig. 1). In total, among 387 whole-slide images, 68, 51, and 85 areas of squamous, Barrett’s, and dysplastic tissue were annotated, respectively. The whole-slide images were then divided into 1000- × 1000-pixel patches, curated to remove patches with limited utility (see supplemental materials for additional pre-processing steps). These curated and labeled patches represent the ground truth for this model, allowing for the calculation of performance metrics. In total, the data were split into a training set and a test set at the patch level (i.e. not at the patient level), with the training set contained 2,849 labeled patches and 889,208 unlabeled patches, and the test set contained 2,645 labeled patches (the model was blinded to these labels) (Table S1). In the same fashion as with pCLE frames, we augmented the labeled and unlabeled datasets by performing horizontal and vertical rotation transformations. Slides stained with H&E are subject to subtle but significant differences in coloration depending on their age and the specific facility in which they were processed. In order to ensure that the model learns based on tissue structure rather than these color variations, we color-normalized the patches by converting them digitally to grayscale using the standard grayscale transformation as follows: gray_pixel = 0.114 * blue_pixel + 0.299 * red_pixel + 0.587 * green_pixel. As the samples collected for this study came from the same institution and were collected within a short time frame, the effect of color variations was subtle and required only the standard RGB-to-grayscale transformation.

#### Esophageal biopsy image classification models

##### Patch-level model

A patch-level model accomplishes two primary goals. First, it aims to classify 1000- × 1000-pixel patches without relying on a human to label hundreds of thousands of images. Second, such a model is capable of producing useful Grad-CAMs, allowing for insight into which features of the image were important to the model. A whole-slide model (as outlined below) cannot accomplish this task. Given the sheer number of patches extracted from the whole-slide images, we opted for a patch-level model that could be trained using a relatively small number of labeled data and a large number of unlabeled data. This approach is known as semi-supervised learning, and our specific approach known as MixMatch^26^. During the training process, two distinct processes, “pseudo-labeling” and “MixUp,” iteratively organize the unlabeled samples and cluster them with similarly labelled samples. The effect results in a method that creates an “illusion” of training on a fully-annotated dataset. Further details of this method are outlined in the Supplemental Methods section (Fig. S2). In order to cross-validate this model, we designated a fixed test set of patches and split the training set (consisting of both labeled and unlabeled patches) into five randomized groups. Each of these five training sets was used to train the model and then test against the fixed test set in order to demonstrate that the model is able to learn effectively across multiple training sets.

##### Esophageal Whole-Slide-Image-Level Model

In addition to a patch-level model, we also developed a model based on a convolutional auto-encoder that is capable of classifying the entire whole-slide image rather than independent patches, more closely aligning with how a human pathologist diagnoses a slide. Such models have shown success in other applications such as breast malignancy^27^, but to our knowledge this work is the first to apply them to whole-slide image classification in esophageal dysplasia.

The whole-slide classification model based on a deep convolutional auto-encoder^5^ was designed as a two-step clustering process in order to decrease the dimensionality of whole-slide images by extracting key features and preserving core information. In the first step, the whole-slide images were encoded as a histogram by applying a combination of an autoencoder and a clustering algorithm. In the second step, a classification model was trained on the encoded whole-slide images. The whole-slide images that were used to both train the autoencoder and construct the clusters in the first step were not used in the second step (training the classifier). A total of 387 whole-slide images from 130 unique patients were collected, with the number of whole-slide images increased to 650 after pre-processing and cropping. From there, 115 whole-slide images from 10 patients were selected randomly to train the autoencoder to extract patch-level image features in the first step, and the rest of the dataset (535 whole-slide images from 120 patients) was used for training and evaluation of classification in the second step. After encoding these 535 whole-slide images, we employed five-fold cross-validation in which, in each fold, images from 24 patients were used as the test set with the images from the remaining patients as the training set. Further details of this method are described in the Supplemental Methods.

#### Esophageal biopsy model visualization

As with pCLE analysis, visualization of the model’s output is crucial in order to determine the tissue structures that the model used for its decision-making and ultimately to obtain physician trust in these deep learning approaches. We extracted tissue feature activation heat maps – Grad-CAMs – by applying an established deep learning methodology^25,29^. We extracted Grad-CAMs at the patch-level in order to visualize areas of the patch that were of the highest relevance to the model.

#### Ethical considerations

This study was approved by the Hunter Holmes McGuire Veterans Affairs Medical Center Institutional Review Board and the University of Virginia Institutional Review Board for Health Science Research (IRB-HSR #21,328). All human tissue samples in this study were obtained in the two prior studies referenced above^6,23^, and all samples were obtained with patients’ informed consent in accordance with the Declaration of Helsinki.

#### Statistical analysis

Statistical analysis was performed using MiniTab Express (version 1.5.3, MiniTab, LLC). Continuous data were compared with two-sample t tests and one-way ANOVA. Categorical variables (e.g. demographic data such as gender ratios) were compared with two-way proportion tests. Differences between variables with a p-value < 0.05 were considered significant. The pCLE models were developed using Tensorflow version 1.14. The biopsy models were performed using Pytorch version 1.0.0 and Torchvision version 0.2.2. The primary programming language used was Python.

## Results

### Patient population

This study included 387 whole-slide images from 130 patients with biopsy data and 79 patients with pCLE data, and the patients were split into multiple individual datasets (as outlined below) with comparable basic characteristics (Table 1). For the biopsy models, labeled train and test sets comprise a relatively small number of labeled patient data compared to the total (26 of 130 patients). The data from the remaining 104 patients were used in the unlabeled training set for the patch-level semi-supervised learning model. Additional details and prior analyses are published in previous work^6^.

### Deep learning models differentiate pCLE videos of squamous tissue, non-dysplastic BE, and dysplasia

**Figure 2:**
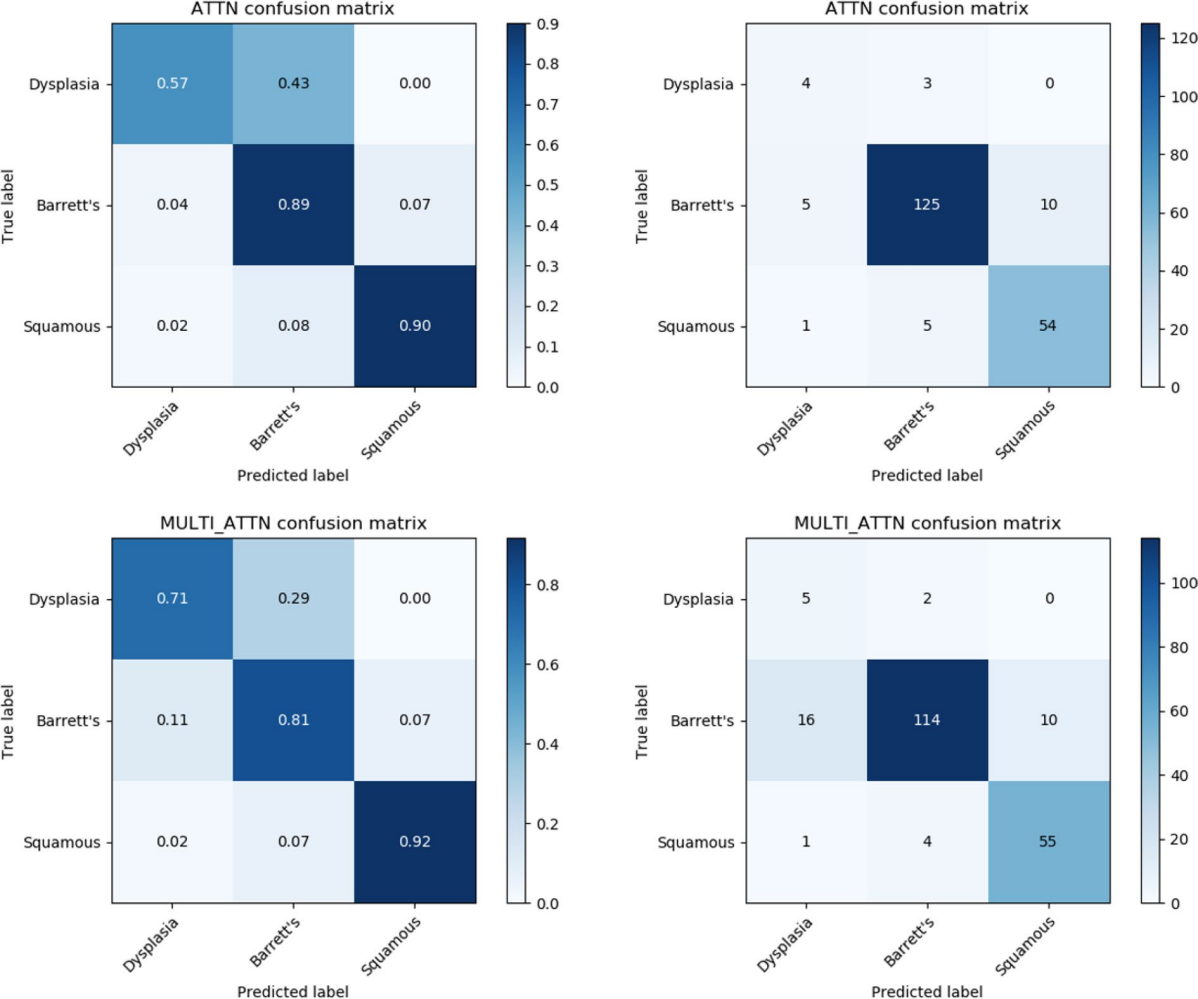
Confusion matrices for the two pCLE models (Attn and MultiAttn) designed to detect dysplasia given its relative rarity. Attn (top left, top right) and the MultiAttn (bottom left, bottom right) models for pCLE classification. The matrices on the left represent normalized data in the form of percentages (e.g. the MultiAttn model correctly classified 71% of true dysplasia sequences). Darker colors indicate higher percentages in a given square. The matrices on the right represent the same data in the form of raw numbers of sequences in each class. Darker colors indicate higher numbers of sequences. The top-left to bottom-right diagonal represents accordance between the true classification and the classification predicted by the model. Created using matplotlib 3.3.2 (https://matplotlib.org/).

**Figure 3:**
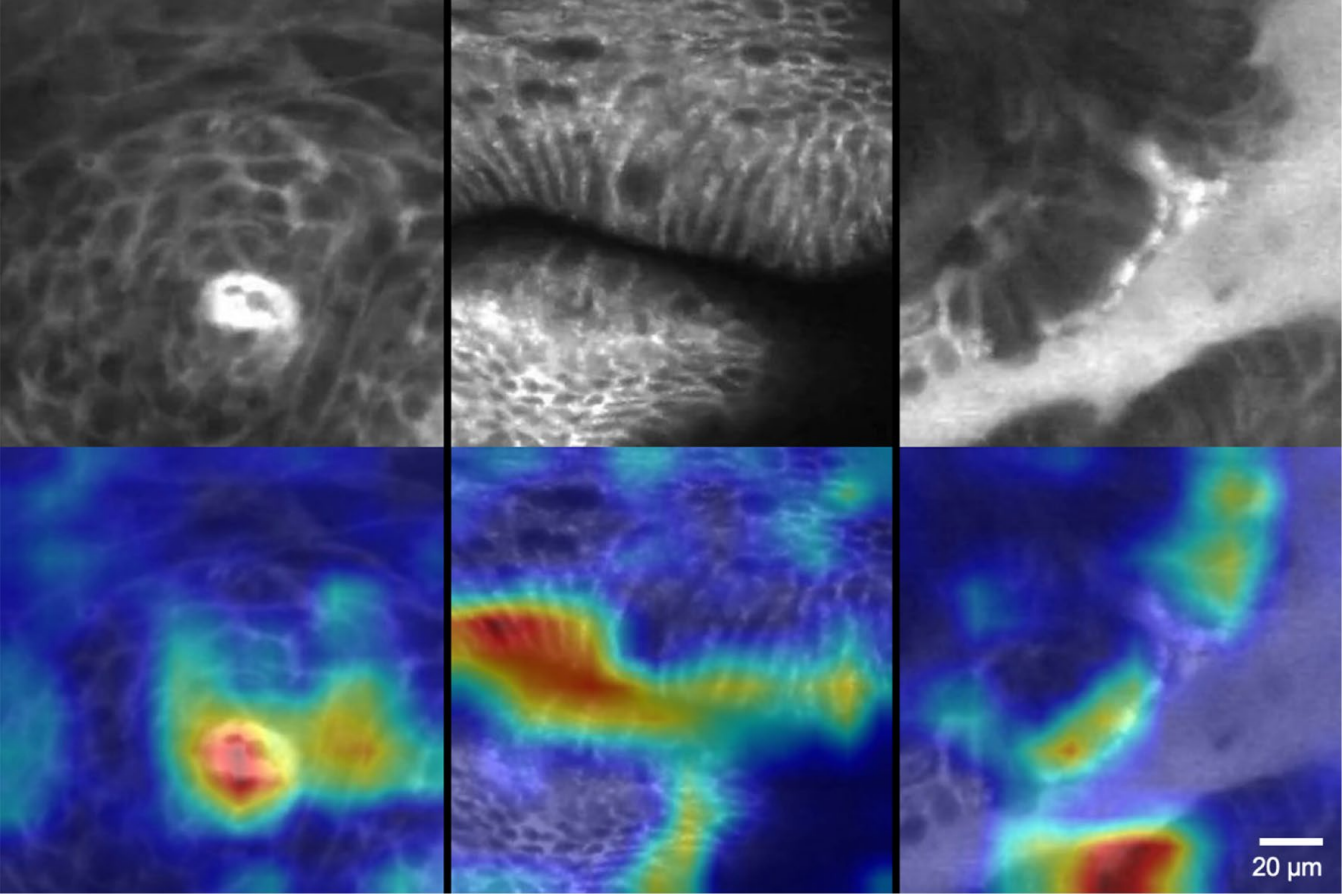
Examples of pCLE images from each class (top row) and gradient-weighted Class Activation Maps (Grad-CAMs) that highlight regions of pCLE frames relevant to classification by the convolutional neural network models (bottom row). See 20-micron reference bar for resolution (bottom right). Red/orange coloration indicates high activation while blue/green coloration indicates low activation. Left column: an example of squamous tissue with the Grad-CAM clearly focusing on the intrapapillary loop in the center as the region of interest. Middle column: an example of Barrett’s esophagus with the goblet cells and columnar epithelium being highlighted as the regions of interest. Right column: an example of dysplasia with the Grad-CAM focusing on the poorly organized, saw-toothed epithelium without clear goblet cells that is indicative of dysplasia. Created using matplotlib 3.3.2 (https://matplotlib.org/).

Our pCLE-based models were deployed to classify pCLE videos as representing squamous tissue, non-dysplastic BE, or dysplasia/cancer. The classification performances for the two pCLE models designed to detect dysplasia given its relative rarity in the dataset – the Attn and MultiAttn models – are outlined in Table 2 and Fig. 2. Both Attn and MultiAttn had high specificity in diagnosing all three classes of tissue (weighted averages of 90% and 92%, respectively). MultiAttn demonstrated a much higher sensitivity for dysplasia (71%) compared to Attn (57%). Overall, the Attn model performed well with a weighted average accuracy of 96% for all classes compared to MultiAttn’s slightly lower average accuracy of 91%. Visualization of the models’ output via Grad-CAMs (Fig. 3) demonstrates that the models are capable of identifying medically relevant structures in each class, such as intrapapillary loops in squamous tissue or saw-toothed epithelium in dysplastic tissue (see Table 3 for additional dysplasia criteria).

**Table 2:** Performance metrics for deep learning models, Mean (95% CI) if applicable.

Modality	Model	Specificity	Sensitivity	PPV	NPV	Accuracy	F1 Score
**pCLE**							
**Attn**							
	Dysplasia	97%	57%	40%	98%	96%	47%
	Barrett's	88%	89%	94%	80%	89%	92%
	Squamous	93%	90%	84%	96%	92%	87%
	Weighted Average	90%	88%	89%	85%	90%	89%
**MultiAttn**							
	Dysplasia	92%	71%	23%	99%	91%	34%
	Barrett's	91%	81%	95%	70%	85%	88%
	Squamous	93%	92%	85%	96%	93%	88%
	Weighted Average	92%	84%	89%	79%	87%	86%
**Biopsy**							
**Patch-level**							
	Dysplasia	89% (85–93)	72% (61–83)	31% (25–37)	98% (97–99)	88% (84–91)	43% (38–47)
	Barrett's	91% (89–93)	81% (74–88)	91% (89–92)	82% (77–88)	86% (83–89)	85% (82–89)
	Squamous	100% (100–100)	92% (91–93)	99% (98–99)	94% (93–95)	96% (95–97)	95% (94–96)
	Weighted Average	93% (91–95)	82% (75–88)	74% (76–90)	92% (89–94)	90% (87–92)	74% (71–77)
**Whole-slide-image-level**							
	Dysplasia	96% (92–100)	90% (79–100)	85% (58–100)	93% (80–100)	93% (90–97)	85% (70–100)
	Barrett's	93% (87–99)	94% (88–100)	86% (66–100)	94% (85–100)	93% (89–96)	89% (78–100)
	Squamous	100% (100–100)	97% (95–99)	100% (100–100)	99% (98–100)	99% (99–100)	99% (97–100)
	Weighted Average	97% (95–99)	93% (89–96)	94% (93–96)	92% (84–100)	94% (92–97)	93% (91–95)
	$Specificity = \frac{TN}{{\left( {FP + TN} \right)}}$ $Sensitivity = \frac{TP}{{\left( {TP + FN} \right)}}$ $PPV = \frac{TP}{{\left( {TP + FP} \right)}}$			$NPV = \frac{TN}{{\left( {TN + FN} \right)}}$ $Accuracy = \frac{{\left( {TP + TN} \right)}}{{\left( {TP + FP + FN + TN} \right)}}$ $F1 = 2{* }\frac{PPV*Sensitivity}{{\left( {PPV + Sensitivity} \right)}}$			*TP* = True Positive *FP* = False Positive *TN* = True Negative *FN* = False Negative PPV = Positive Predictive Value NPV = Negative Predictive Value

**Table 3:** Criteria for the diagnosis of dysplasia using probe-based confocal laser endomicroscopy (pCLE) and hematoxylin-and-eosin-stained biopsies.

pCLE Criteria for Dysplasia^31,33^	Biopsy Criteria for Dysplasia^34^
Enlarged, irregular cells	Distorted gland architecture
Glands vary in size and spacing	Gland crowding
Crowded glands	Pleomorphism
Variable epithelium thickness	Enlarged nuclei
Saw-toothed epithelium	Mitotic activity
Relative lack of goblet cells	Hyperchromasia

### Deep learning models differentiate biopsies of squamous tissue, non-dysplastic BE, and dysplasia at two magnification levels

#### Biopsy model performance at the 1000- × 1000-pixel patch level

**Figure 4:**
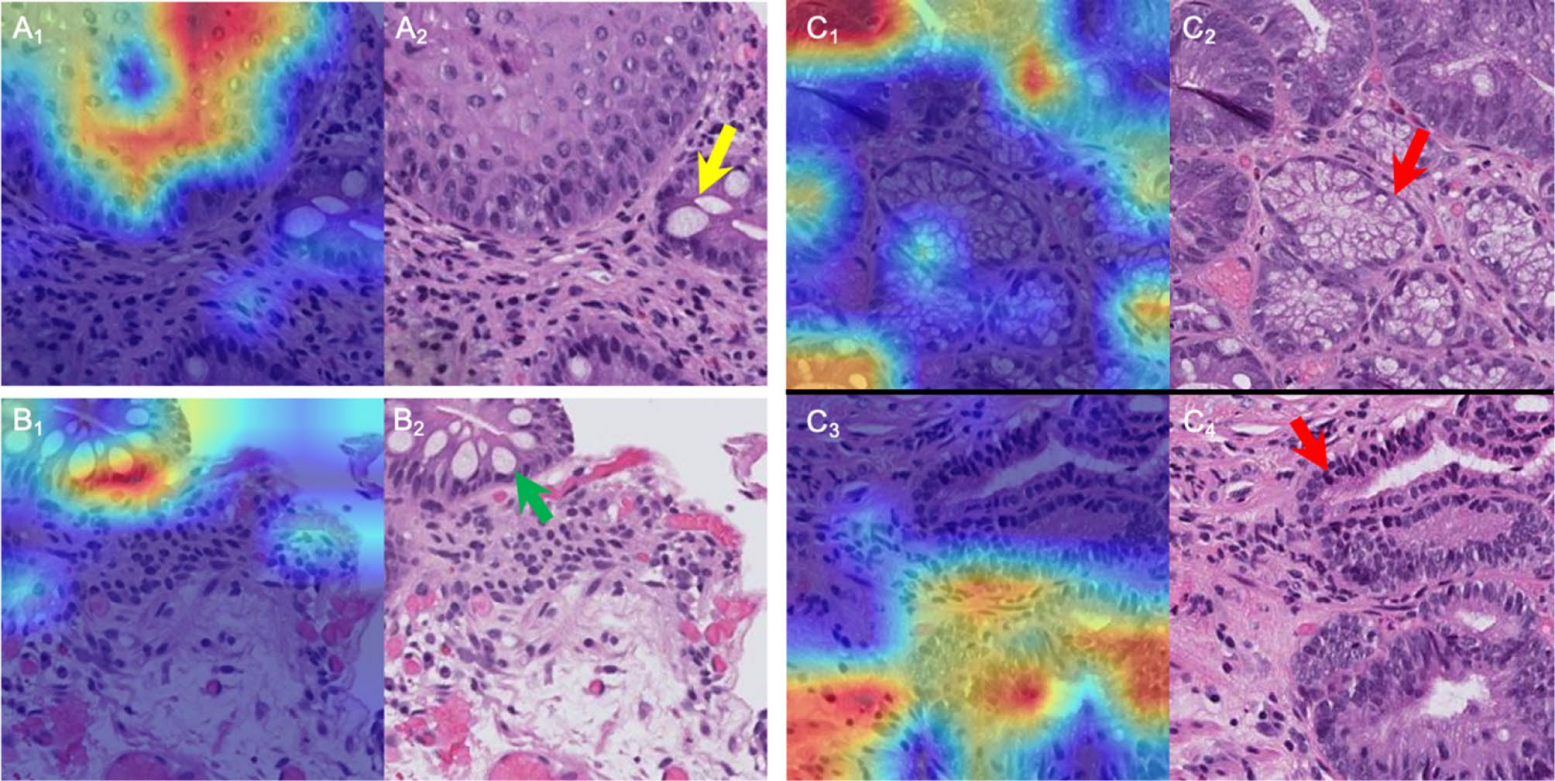
Gradient-weighted Class Activation Maps (Grad-CAMs) that highlight regions of biopsy images that are relevant to classification by the deep learning model (hematoxylin and eosin stain, 40 × magnification). Red/orange coloration indicates high activation while blue/green coloration indicates low activation. Created using matplotlib 3.3.2 (https://matplotlib.org/). (**A**) Grad-CAM (A_1_) and corresponding biopsy patch (A_2_) for an example of squamous tissue abutting intestinal tissue. The model clearly shows the highest activation in the squamous area while showing lower activation in the surrounding intestinal-like tissue with visible goblet cells (yellow arrow). (**B**) Grad-CAM (B_1_) and corresponding biopsy patch (B_2_) for an example of non-dysplastic Barrett’s. Here, the model shows the highest activation in the intestinal-like tissue with visible goblet cells (green arrow) and low activation in the surrounding connective tissue. (**C**) Grad-CAMs (C_1_ and C_3_) and corresponding biopsy patches (C_2_ and C_4_) for examples of dysplasia. Note that the regions of high activation are focused on areas of nuclear atypia and hyperchromasia while ignoring clearly non-dysplastic tissue (red arrows).

Table 2 summarizes the results of the patch-level model designed to differentiate between our three tissue types of interest using 1000- × 1000-pixel patches that represent small portions of whole-slide images. Our results demonstrate that the model can capably distinguish between normal squamous tissue, non-dysplastic BE, and dysplasia. The mean overall accuracy of the model is 90% (95% CI, 87–92%), and for dysplasia in particular it performs well with a mean sensitivity of 72% (95% CI, 61–83%) and specificity of 89% (95% CI, 85–93%). Grad-CAMs for the three different classes demonstrate that the patch-level model is capable of highlighting features relevant to each class (Fig. 4). For example, Fig. 4C shows dysplasia Grad-CAMs with high activation in areas of pleomorphism and hyperchromasia (see Table 3 for additional dysplasia criteria and Table S2 for additional Grad-CAM examples for each class) and, importantly, low activation in areas that clearly do not represent dysplasia, such as connective tissue and nondysplastic BE.

#### Biopsy model performance at the whole-slide-image level

Table 2 summarizes the results of the whole-slide-image-level model, which demonstrate that this model can also distinguish between normal squamous tissue, non-dysplastic BE, and dysplasia. The overall accuracy of the model is slightly higher than the patch-level model at 94% (95% CI, 92–97%). For dysplasia in particular it performs well with a sensitivity of 90% (95% CI, 79–100%) and specificity of 96% (92–100%), but the confidence intervals tend to be wider given the relatively small number of whole-slide images compared to patches.

## Discussion

Diagnosis of BE with and without dysplasia is critical to the prevention and management of esophageal malignancy, and the current state of diagnostic methodology leaves significant room for improvement. The goal of this investigation was to train deep learning models to classify both pCLE videos and whole-slide biopsies to model the gold standard of histopathological diagnosis^4^. Our deep learning models for pCLE achieved sensitivities for dysplasia as high as 71% with accuracy for dysplasia above 90%. In previous studies, reported human real-time pCLE sensitivity compared to pathological diagnosis is 60% for low-grade dysplasia and 67% for high-grade dysplasia/cancer, suggesting that our deep-learning-based models are indirectly comparable with human interpretation, although direct comparisons are needed to demonstrate this more clearly^6^. While these results are noteworthy of their own accord, one major issue plaguing the use of artificial intelligence in medicine is the so-called “black box” of deep learning, an analogy which describes the lack of insight that humans have into how the models arrive at their decision-making^30^. Our effort to extract Grad-CAMs from our pCLE models helps illuminate this black box by demonstrating exactly which tissue structures the model uses to make its decisions. Our model’s predictions tend to be based on the same criteria that humans use for pCLE-based diagnosis, such as glandular crowding and pleomorphism to detect dysplasia, as demonstrated qualitatively by the selected Grad-CAMs. These findings may open the road for increased utilization of pCLE in bedside applications without requiring an expert interpreter, something currently under-investigated in the field of artificial intelligence in medicine^30^.

In an attempt to model the gold standard of histopathological diagnosis^4^, our biopsy-based deep learning models achieved an overall accuracy approaching that of expert humans as reported in previous studies^31^. We achieved superb specificity for all classes (squamous, non-dysplastic Barrett’s, and Barrett’s esophagus with dysplasia) and reasonably high sensitivity for dysplasia. In practice, high sensitivity for dysplasia translates to reduced risk of missed dysplasia progressing to cancer^5,9^. However, the low positive predictive values and high negative predictive values for dysplasia with both the pCLE models and the patch-level biopsy suggests that, in practice, these would perhaps be a more useful tool for ruling out suspected dysplasia rather than detecting dysplasia in non-suspicious areas. The whole-slide image model appeared to have somewhat higher sensitivity and positive predictive value for dysplasia, but relatively wide confidence intervals produced during the cross-validation suggest that superiority of this model cannot be concluded with this small dataset.

As was the case with pCLE, utilizing deep learning to highlight areas of interest on biopsy images in the form of Grad-CAMs illuminates the otherwise black box of deep learning and provides much needed “explainability” for clinicians and researchers. Our Grad-CAMs support the idea that the model is discovering for itself the features that define each class of tissue. Continued improvements to these models may be able to sub-classify dysplasia as indefinite, low-grade, or high-grade, a notoriously tricky distinction to make in esophageal neoplasia. Grad-CAMs focused on this task may then be able to draw attention to previously unknown distinctions between the two severity subclasses of dysplasia and aid human pathologists’ in their diagnosis.

With regards to the multimodal nature of the study, which explored two pCLE video models as well as two distinct methods for biopsy analysis with deep learning, the goal was to present methodologies for classifying esophageal tissue using both minimally invasive (i.e. pCLE) and more traditionally invasive (i.e. biopsy) modalities. Our results suggest that each modality has its strengths and limitations. For example, pCLE video analysis avoids the need for biopsy and could potentially be employed in real time during an endoscopy, which would not be possible for biopsy. However, its sensitivity for dysplasia is lower than the biopsy models, whole-slide image in particular, and it may be better suited for identifying Barrett’s in an at-risk individual presenting for first-time screening. When comparing biopsy models, our study also suggests that the whole-slide image biopsy analysis may have higher sensitivity for dysplasia in particular, but as each data point is an entire whole-slide image, it requires substantially larger datasets than the patch-level model in order to achieve narrow confidence intervals during validation. The multimodal nature of this study demonstrates proof of concept for each of these methods, opening the door for further exploration of each.

Major strengths of our study include the relatively large volume of data used to train the models, particularly in terms of patch-level biopsy analysis, and the wide variety of performance metrics (e.g. accuracy, sensitivity/specificity, PPV/NPV, F1 score) used to assess the validity of said models, something often missing from work done on deep learning in medicine^30^. Typically, large volumes of data involve subsequently high time and effort costs to interpret the data. An additional strength of our study is that our biopsy classification tools used novel deep learning annotation augmentation techniques to minimize the amount of upfront annotation by clinicians and researchers. Moreover, our use of Grad-CAMs as heat maps for important tissue features will help increase user confidence in artificial-intelligence-based decision-making. The value of such an explainable artificial-intelligence-based diagnostic tool would be to provide opportunity for physicians undertrained in pCLE to use it with confidence rather than requiring intensive training in pCLE interpretation^6^.

However, despite these strengths, we experienced limitations as well. Our primary limitation was the availability of dysplasia data, particularly for pCLE video sequences. This paucity of data also limited our ability to perform cross-validation studies for the pCLE results as we did for the biopsy models, limiting the generalizability of our pCLE results. In order to more definitively demonstrate the performance of such deep learning models, future studies will require larger and more balanced datasets. Although in reality, diseased tissue will always be less common than healthy tissue, training deep learning models on large, balanced datasets will allow for improved testing performance on imbalanced test sets that better reflect the real world. Our study design also does not account for direct comparisons of human interpreters to our deep learning models, meaning that our comparisons to human results are indirect. Additionally, gastric tissue was not included in our analysis, somewhat limiting the ability of these models to be applied to any esophageal biopsy. Moreover, examples of tissue labeled as either “intestinal metaplasia” or “dysplasia” that appeared to be borderline or indefinite were excluded from the models’ training and testing sets in order to provide the clearest examples of each class as inputs for the model. In reality, these classes are not distinct and exist on a spectrum. Identifying dysplasia is further clouded by the fact that inflammation of the esophagus shares many features with dysplasia^32^, and our models did not account for these changes. Future analyses should aim to include even larger datasets, allowing for the incorporation of more ambiguous examples in order to hone the model’s accuracy and clinical utility, a task that is made easier using our specific approaches.

## Conclusion

Deep learning models represent an exciting opportunity to improve upon the gold standard of diagnosis of esophageal dysplasia and its precursors, both in the form of classic histopathological diagnosis as well as by novel technologies such as pCLE. Our work indirectly suggests that these models may achieve similar accuracy as human interpreters, paving the way first for more direct comparisons of humans and deep learning models and then for increased clinical application of these technologies. Further work in this area will advance the clinical use of deep learning in medicine, improving the effectiveness and efficiency of patient care in the field of gastroenterology and beyond.

## Supplementary Information

Supplementary Information.
