# Supplementary material for: Deep learning systems detect dysplasia with human-like accuracy using histopathology and probe-based confocal laser endomicroscopy
Source: Sci Rep. 2021 Mar 3;11:5086. doi: 10.1038/s41598-021-84510-4 (PMC7930108; doi:10.1038/s41598-021-84510-4)
Supplement: Supplementary file 1 — Supplementary Information. [file 41598_2021_84510_MOESM1_ESM.docx]

**Title:** Deep learning systems detect dysplasia with human-like accuracy using histopathology and probe-based confocal laser endomicroscopy

Shan Guleria, MD^1^  | Tilak U. Shah, MD^2,3^  | J. Vincent Pulido, MS^4^

Matthew Fasullo, MD^2,3^ | Lubaina Ehsan, MBBS^5^  | Robert Lippman, MD^2^

Rasoul Sali, MS^6^ | Pritesh Mutha MD, MPH^2,3^ | Lin Cheng, MD, PhD^1^ | Donald E. Brown, PhD^6^ | Sana Syed, MD, MS^5^

^1^ Rush University Medical Center, Chicago, IL

^2^ Hunter Holmes McGuire Veterans Affairs Medical Center, Richmond, VA

^3^ Virginia Commonwealth University, Division of Gastroenterology, Hepatology and Nutrition, Richmond, VA

^4^ Johns Hopkins University Applied Physics Laboratory, Laurel, MD

^5^ University of Virginia School of Medicine, Charlottesville, VA

^6^ University of Virginia, Department of Systems & Information Engineering, Charlottesville, VA

Corresponding Author: Sana Syed, MD, MS*

*Correspondence to ss8xj@virginia.edu

**Supplemental Materials**

**Methods**

pCLE Video Model Design

**pCLE Datasets**

In order to decrease the memory requirements of the network, the data were down-sampled by using every tenth frame from each sequence, reducing them from 120 frames to 12. We also resized the frames to a height and width dimension of 224 × 224 pixels. To ensure the model only learns from the pertinent portions of the video, irrelevant portions were removed (e.g. commercial logos, blank space outside the scope of the lens, etc.). The training set was augmented by randomly flipping and performing 0-, 90-, 180-, or 270-degree rotations on each individual frame in a clip independently.

**pCLE Video Classification Models**

We developed a 3-tiered model to process pCLE videos^20^. A sequence of videos was presented to the model as individual frames. Each frame of a given video was then processed using a frame-level network producing frame representations. These representations were aggregated by a network to produce video representations which are then used to classify videos using the classifier network. We utilized two types of network: one using a traditional attention layer (Attn), and another using class-specific multi-module attention layers (MultiAttn). Each model was trained end-to-end with 32 epochs using an Adam optimizer. For complete details of the pCLE video models, see Figure S1 for a block diagram with the general model architecture as well as our group’s methods paper on this subject^20^.

**Biopsy Dataset Pre-processing**

The whole-slide images were patched at the 40x magnification level using a sliding window approach with a window size of 1000x1000 pixels. There was a 500-pixel overlap between patches. White space in each patch was measured using a binary thresholding method: if 45% or more of the grayscale image is white space (greater than pixel value of 125), then the patch was discarded from the dataset due to excess white space. The data were then augmented using vertical and horizontal flips randomly and uniformly random zoom scaled between 0.8 and 1.0.

**Biopsy Image Classification Models**

*Patch-level Model*

We implemented a state-of-the-art technique for semi-supervised learning called MixMatch. Although simple to implement, it has achieved noteworthy results on benchmark computer vision datasets^25^. During the training process, two distinct processes, “pseudo-labeling” and “MixUp,” iteratively organize the unlabeled samples and cluster them with similarly labelled samples. The effect results in a method that creates an “illusion” of training on a fully-annotated dataset.

MixMatch Methods Details

The MixMatch^25^ method can be broken down into two parts: **Pseudo-labeling** (initially guessing labels) and **MixUp**^34^ (generating synthetic labeled data). One key advantage of this method is that it can be applied to any standard classification model. An algorithm map is provided (Figure S2).

Pseudo-label

MixMatch attempts to first guess the labels of samples lacking annotations by averaging the probabilities as various transformations are applied to a patch (e.g. horizontal and vertical rotations). This average probability score is then accentuated using a sharpening procedure where we increase the score of the higher class probabilities and dampen the scores of the lower class probabilities:

$$\bar{y}_{ave}= \frac{1}{K}\sum_{\left\{ k=1 \right\}}^{K} p(u)$$

$$\hat{y_{i}}=\frac{\left( \bar{y}_{ave,i} \right)^{\frac{1}{T}}}{\sum_{j=L}^{L} \left( \bar{y}_{ave,j} \right)^{\frac{1}{T}}}$$

The intuition here is that if the model, on average, finds that a patch is a certain class then the best guess label of this patch is the class with the highest probability. Sharpening this score increases the confidence that a patch belongs to a certain class.

MixUp

MixUp^3^ is the process of generating more synthetic data by performing a pixel level interpolation between images and pairwise interpolation between probability distribution. For a pair of two examples with their corresponding labels probabilities $\left( x_{1},y_{1} \right)$ and $(x_{2}, y_{2})$, we compute $(x', y')$ by:

$$\lambda\sim Beta(\alpha, \alpha)$$

$$\lambda'\sim\max\left( \lambda, 1-\lambda\right)$$

$$x^{'}=\lambda^{'}x_{1}+\left( 1-\lambda^{'} \right)x_{2}$$

$$y^{'}=\lambda^{'}y_{1}+\left( 1-\lambda^{'} \right)y_{2}$$

$\lambda$ is a Beta distribution governed by the distribution's parameter $\alpha$. MixUp is applied between any randomly chosen pairs of training samples (labeled or unlabeled).

$$W=X\cup U$$

$$X^{'}=MixUp(X, W)$$

$$U^{'}=MixUp(U, W)$$

The synthetically generated samples of labeled, $X'$, and unlabeled, $U'$, data are presented to the model for training.

MixMatch treats the labeled and unlabeled data differently. For all labeled samples, $X$, the loss is computed using a standard cross-entropy loss:

$$L_{X}=-\sum_{x\in X} y*\log p\left( x \right)$$

where $y$ is the ground truth binary indicator if the class label is the correct classification for observation $x$, and $p$ is the probability output for each class.

For unlabeled samples, $U$, the loss is a mean square error:

$$L_{U}=\sum_{u\in U} \left| \left| \hat{y}-p\left( u \right) \right| \right|^{2}$$

The total loss becomes:

$$L=L_{X}+\lambda_{U}L_{U}$$

*Whole-Slide-Image-Level Model*

The whole-slide classification model based on a deep convolutional auto-encoder^5^ was designed as a two-step clustering process in order to decrease the dimensionality of whole-slide images by extracting key features and preserving core information. In the first step, the whole-slide images were encoded as a histogram by applying a combination of an autoencoder and a clustering algorithm. We used ResNet18 as an encoder to map image patches to an embedding space, with the dimension of the embedding vector extracted from each patch was 1024. The decoder was comprised of convolutional and up-sampling layers in order to increase the size of the feature map and to restore the original size of the input image.

In the second step, a classification model was trained on the encoded whole-slide images. The whole-slide images that were used to both train the autoencoder and construct the clusters in the first step were not used in the second step (training the classifier). In the second step Gaussian Mixture Model (GMM)^6^ initialized by k-means^7^ was applied to cluster the extracted features into 120 clusters. Constructed clusters are representative of key features. For feature extraction we did not used any information regarding labels of whole-slide images. To patching whole-slide images, a sliding window method was applied to each high-resolution whole-slide image to generate patches of size 128 × 128 pixels.

A total of 387 whole-slide images from 130 unique patients were collected, with the number of whole-slide images increased to 650 after pre-processing and cropping. From there, 115 whole-slide images from 10 patients were selected randomly to train the autoencoder to extract patch-level image features in the first step, and the rest of the dataset (535 WSIs from 120 patients) was used for training and evaluation of classification in the second step.

These processes were used to encode 535 whole-slide images for slide-level classification. After patching the whole slide image $I_{i},i=1,2,\ldots,535,$ the auto-encoder trained in previous step was employed to map each patch $I_{ij}$ to embedding space, $e_{ij}$. Then posterior probability of each component of GMM $z_{k},k=1,2,\ldots,120$ given the data, $p\left( z_{k} | e_{ij} \right),$ were computed. This process encodes each patch to a vector with 120 elements. Finally, summation over vectors of all patches belong to whole-slide image $I_{i}$ followed by normalization creates a representative vector for use in training and validation of the whole-slide-image-level classifier.

After encoding whole slide images, they were used to train and evaluate a whole-slide-image-level classifier. A support-vector machines classifier was trained to categorize whole-slide images into three classes: Dysplasia, Barrett’s, and Squamous. We employed a five-fold cross-validation as outlined in the main manuscript in order to validate the results and provide means and standard deviations.

*Table S1. Breakdown of the class split and the training/validation/testing split for the patch-level biopsy and pCLE classification models.*

| **Patch-Level Biopsy Model** | | | | |  |
| --- | --- | --- | --- | --- | --- |
|  | Labeled Train  (% of Total Labeled) | Test  (% of Total Labeled) | **All Labeled**  **(% of Total Labeled)** | Unlabeled Train** | |
| Squamous | 1,308 (24%) | 1,121 (20%) | **2,429 (44%)** | - | |
| Barrett's | 925 (17%) | 1,365 (25%) | **2,290 (42%)** | - | |
| Dysplasia | 616 (11%) | 159 (3%) | **775 (14%)** | - | |
| **Total** | **2,849 (52%)** | **2,645 (48%)** | **5,494 (100%)** | **889,208** | |
| **The semi-supervised learning method used for this patch-level model allows for a large proportion of the training set to remain unlabeled. The percentage breakdown above represents fractions of the labeled data that belong to each class. | | | | | |
|  |  |  |  |  | |
| **pCLE Models** | | | | |  |
|  | Train (% of total) | Validation (% of total) | Test (% of total) | Total | |
| Squamous | 180 (17.0%) | 60 (5.7%) | 60 (5.7%) | **300 (28.4%)** | |
| Barrett's | 440 (41.6%) | 140 (13.2%) | 140 (13.2%) | **720 (68.1%)** | |
| Dysplasia | 20 (1.9%) | 10 (0.9%) | 7 (0.7%) | **37 (3.5%)** | |
| **Total** | **640 (60.5%)** | **210 (19.9%)** | **207 (19.6%)** | **1057 (100%)** | |

*Table S2. Grad-CAM Examples by Tissue Type.*

| Tissue Type | Feature | Example Grad-CAM |
| --- | --- | --- |
| Squamous | Nonkeratinized stratified squamous epithelium | 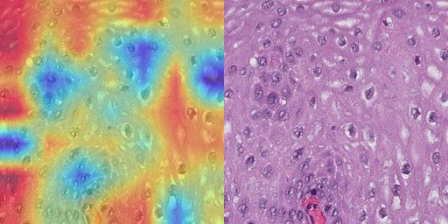 |
|  | Nonkeratinized stratified squamous epithelium with distinct layer of basal cells (yellow arrow) | 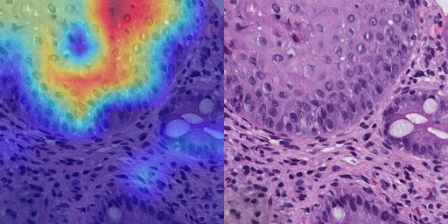 |
| Non-dysplastic Barrett’s Esophagus | Abundant goblet cells | 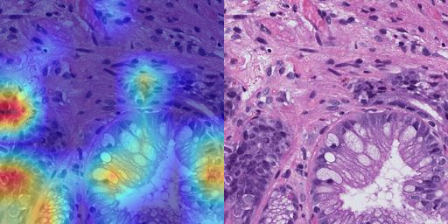 |
|  | Well-organized glands with regular nuclei  Low nucleus:cytoplasm ratio | 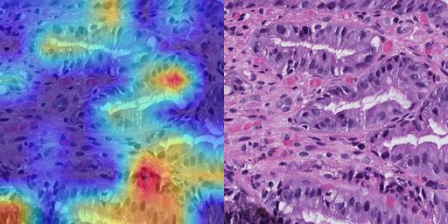 |
| Dysplasia | Crowded glands  Glandular distortion | 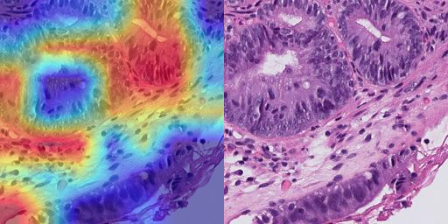 |
|  | Pleomorphism  Hyperchromasia  Open chromatin pattern  Increased nucleus:cytoplasm ratio | 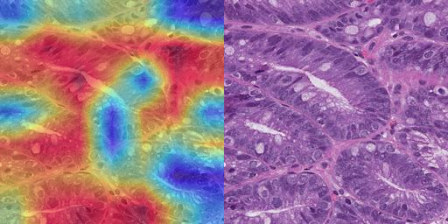 |

*Examples of gradient-weighted class activation maps (Grad-CAMs) for each tissue type alongside corresponding biopsy patches.* *The Grad-CAMs are on the left side of each pair and the original biopsy image is on the right (hematoxylin and eosin stain, 40x magnification). Red/orange coloration indicates high activation while blue/green coloration indicates low activation. The Grad-CAMs demonstrate areas of relevance to the deep learning model when making its classification. These examples show that the model is activated by common pathologic features of each class^8^. Created using matplotlib 3.3.2 (https://matplotlib.org/).*

**Figure S1:** A block diagram representing the pCLE network employed. The general architecture of the model consists of three sequential modules. First, the frame-level network converts video frames into a 256-dimensional frame representation via five convolutional groups of two two-dimensional convolutions, a batch-norm layer and a max-pool layer. Second, the pooling network aggregates these frame-level representations into a video-level representation, labeled R (this is where the Attn and MultiAttn models were employed). Lastly, the video-level representation was run through the classifier module, consisting of a dense layer and a softmax operation, in order to obtain probabilities of the video sequence belonging to one of the three tissue classes.


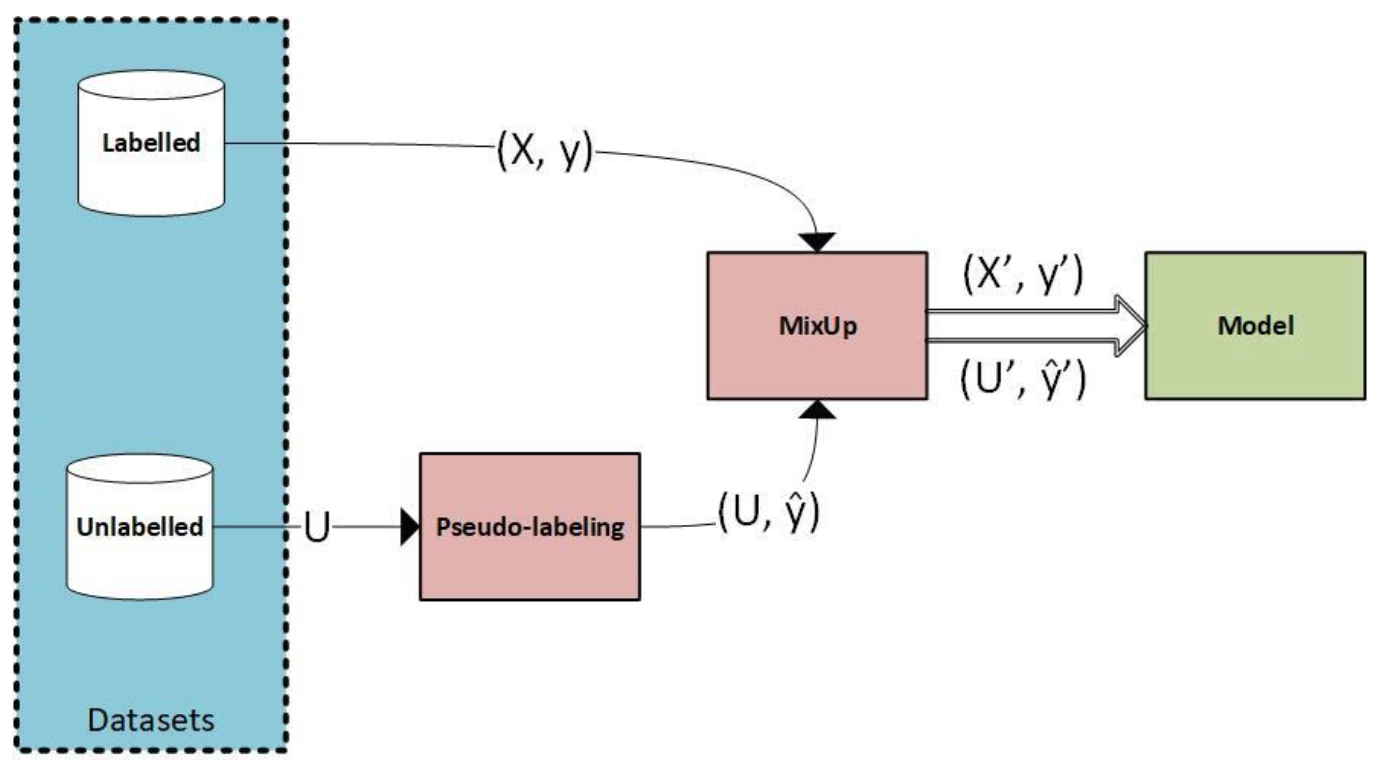


**Figure S2:** Summary of MixMatch method. The labeled and unlabeled datasets are treated differently. Unlabeled datasets are first provided pseudo-labels, or best guess labels, using the model during training time. The labeled and unlabeled datasets are then used to create synthetic data to present to the model for training.
